# Reminiscences of an aspiring graduate student in the 1970s who worked on kuru-related projects with Dr Gajdusek

**DOI:** 10.1098/rstb.2008.4005

**Published:** 2008-11-27

**Authors:** Richard Benfante

**Affiliations:** Apt 81210401 Grosvenor Place, Rockville, MD 20852, USA

The story of kuru for me has been an opportunity to work with and know some of the most extraordinary people: Dr Carleton Gajdusek, Dr Vin Zigas and Dr Mike Alpers, to name only a few, and, of course, their many interesting colleagues, families and friends.

To begin: I first heard of kuru *ca* 1960. I was still in high school and was fascinated by many of the interesting facts in the Guinness Book of Records. Kuru was mentioned at the time as being the world's rarest disease, affecting only the Fore people in Papua New Guinea. I was a young graduate student in anthropology the next time I recall this intriguing disease being mentioned. One day in a medical anthropology seminar, the professor was assigning seminar topics for the four persons in the class. Kuru was on the list of topics and the person seated next to me wanted kuru as his topic, as did I. Whether by grace or by chance, the professor called on me first to make a choice, and I chose kuru. That fortuitous event set my life in the right direction. I later wrote my Master's thesis on kuru and began a communication with Dr Gajdusek (Carleton).

The following year, in 1970, I was given an opportunity to work in Carleton's Laboratory at the National Institutes of Health (NIH) in Bethesda, Maryland, initially for approximately 2 years. It was a fortunate coincidence that Dr Vin Zigas and Dr Mike Alpers were also working in the section as visiting scientists.

I remember Vin Zigas, for those of you who may never have met him, as having somewhat of the appearance, as well as the debonair flair, of a Danny Kaye or a Peter O'Toole. But, of course, Vin was unique—a very insightful man with a heart of gold, who lived life as a celebration 24 hours a day. We worked together on several kuru region serology projects. I eventually did my doctoral work on serological and genetic studies on the people of the kuru region and elsewhere in New Guinea.

As for Michael Alpers, it has already been mentioned what a fabulous person and scientist he is. And Dr Gajdusek, what about Dr Gajdusek? What an incredible, pleasant surprise he was. I will mention only a few things, as many of you already know him well—a master of exaggeration and humour in order to make an important point. Here is an example: I remember when Vin Zigas and I were tasked to inventory the New Guinea serum holdings, which covered perhaps 50 storage freezers. I asked Dr Gajdusek why the sera were not stored in walk-in freezers rather than the many Revco and stand-up freezers. He looked up, came out of his concentration on something he was studying and said, somewhat loudly, ‘We tried that at Walter Reed a few years ago and when the giant walk-in freezer failed and then re-froze, we had to hire Everest climbers with buzz saws to rescue the sera from the re-frozen ice.’ ‘Everest climbers with buzz saws’: as far as humorous exaggeration goes, you could hardly improve on that, and he made the point perfectly.

Another time Judy Farquhar and I were tasked to move approximately 100 kuru brains in formalin containers to Dr John Enders' laboratory at Harvard for neuropathological studies. In order to accomplish this, we used a four-wheel station wagon from the NIH motor pool. Carleton somewhat gleefully referred to this situation as providing for ‘a world record brain-to-wheel ratio’.

I never became bored with Dr Gajdusek's superlative sense of humour—it gave his natural genius a human and loving quality and it still does. He used to poke fun at my doing yoga or meditation in the laboratory. However, I remember meditating one early morning in 1976. I was alone in the laboratory, and I believe it was a member of the Swedish embassy who called and told me Carleton had won the Nobel Prize. What a morning and what a day that was! A friend of the laboratory later commented in fun that Carleton may have been the first person to shout his way to the Nobel Prize.

Dr Gajdusek and the people who loved working with him, such as Michael Alpers and Vin Zigas, share some common characteristics that can only inspire the right qualities in the sincere aspiring scientist, namely:

- a reverence and respect for knowledge and a love of truth;
- a willingness to be wrong in order to progress;
- a supportive tolerance of those less learned who are willing and eager to learn.

I am sure one could add other qualities to this list.

Such is my limited selection of sentimental reflections on part of the kuru story and its place in my life. In summary, it was an opportunity to work with extraordinary people in an atmosphere of celebration of love, intelligence and the pursuit of excellence.

